# Preclinical safety and immunological efficacy of *Alternaria alternata* polymerized extracts

**DOI:** 10.1002/iid3.212

**Published:** 2017-12-19

**Authors:** María Morales, María T. Gallego, Victor Iraola, Raquel Moya, Soraya Santana, Jerónimo Carnés

**Affiliations:** ^1^ Research & Development Department Laboratorios LETI Tres Cantos Spain; ^2^ Neuron Biolabs Granada Spain

**Keywords:** allergoid, immunotherapy, mold allergy

## Abstract

**Introduction:**

*Alternaria alternata* is a widespread fungi whose allergy is a risk factor for asthma development. The use of a polymerized allergen extract (allergoid) may be safer than native extract based treatments while maintaining efficacy. The objective of this study was to characterize biochemically and immunochemically a new *Alternaria alternata* allergoid.

**Methods:**

Characterization of native and allergoid extracts was performed by determination of protein content, protein and allergenic profile, biological potency, identification of *Alternaria* allergens, and Alt a 1 quantification. Safety was evaluated in toxicological assays (Ames test, limit test, and fish embryo acute toxicity test in zebrafish, and maximum tolerated dose and Dose‐range finding study in rats). Efficacy was evaluated as the capacity to induce IgG antibodies that block IgE‐binding to the allergen and cytokine induction (IFN‐γ, IL‐4, IL‐6, IL‐10, and TNF‐α) in PBMC from atopic donors.

**Results:**

Protein and antigenic profiles showed significant modification of the depigmented allergoid with respect to the native extract, inducing a lower IgE binding capacity. Alt a 1, Alt a 3, Alt a 6, and Alt a 8 allergen sequences were identified in the polymer. No toxicological nor genotoxicity effects were observed. The polymer induced IgG antibodies that blocked human IgE binding epitopes, and it induced higher IL‐10 levels and similar levels of the other cytokines than native extract in PBMC.

**Conclusions:**

This new *A. alternata* allergoid could be an effective immunotherapy treatment leading to cytokine stimulation and inducing synthesis of IgG antibodies able to block IgE binding to the allergen. In addition, no toxicological effect was observed, and it may be safer than native extract due to its lower IgE binding capacity and cytokine induction that suggest tolerance induction via T cell shift to Treg (IL‐10).

## Introduction

*Alternaria alternata* is a widespread, allergenic saprophyte fungi, usually found on plants, soil, food, and indoor air 1. *A. alternata* spores can be detected from spring to late Autumn in most temperate areas 2, but their levels are especially high in late summer 3, although most patients with fungal allergies have perennial symptoms. Weather conditions such as temperature, relative humidity, and wind speed favor spore releasing dissemination 1, which presents a positive correlation with respiratory symptoms in *A. alternata* monosensitized patients 4, 5.

According to The National Health and Nutrition Examination Survey and The Global Asthma and Allergy European Network, the prevalence of allergy to *A. alternata* is 12.9% in the US and 8.9% in Europe 6, 7. Moreover, *A. alternata* allergy is not only related with typical allergic symptoms like rhinoconjunctivitis but is also a risk factor for developing asthma 8, 9, 10. A positive correlation has been found between spore levels and the occurrence of hospitalization, asthma treatment, and even asthma‐related mortality 11.

Spores are considered the primary source of *A. alternata* allergens, although hyphae, fragmented spores, and dust particles can also release allergens. Until now, 12 different *A. alternata* allergens are reported in the IUIS webpage (http://www.allergen.org/), and five more additional allergens are included in the Allergome (http://www.allergome.org/) database. Alt a 1 is the only major allergen described until now 12, as it is estimated to be recognized by more than 85% of the *Alternaria* allergic population 13. Alt a 1 is a 30 kDa dimer (16.4 and 15.3 kDa‐bands in SDS–PAGE) whose biological function remains unknown, though it may favor fungal colonization, blocking plant defenses 14. Molecular diagnosis to Alt a 1 is used as primary marker of sensitization to *A. alternata* and a significant correlation among Alt a 1 levels and symptoms in monosensitized patients has been described 4. Moreover, other *A. alternata* allergens, considered minor allergens, may have importance in the development of polysensitization, and their role in the induction of IgE‐mediated diseases is not well determined 12.

As a consequence of the clinical implications induced by *A. alternata* 15, the allergen immunotherapy (AIT) to this allergenic source has been considered an appropriate alternative for treating allergy‐suffering patients 16. Until now, the efficacy of *Alternaria* treatments have been demonstrated with native extracts 17, 18 but safety issues are still pending. Another alternative for *Alternaria* immunotherapy is the use of chemically modified allergoids but so far no reports have been published.

In consequence, the objective of this study was to produce and characterize a purified polymerized allergenic extract from *A. alternata* for treating *Alternaria* sensitized patients from an immunochemical and safety perspective. Additionally, we aimed to investigate its immunological activity and its capacity to stimulate a Treg and Th1 response in blood samples from allergic patients.

## Methods

### Allergen extract preparation

Freeze‐dried spores and mycelia from *A. alternata* strain CBS 103.33 (Allergon, Ängelholm, Sweden) were homogenized and extracted in 0.125 M ammonium bicarbonate‐0.15 M NaCl pH 7.5 at 4°C under continuous magnetic stirring obtaining the native extract (NE). The NE was depigmented by mild acid treatment with HCl at pH 2 during 15 min followed by dialysis with a cut‐off of 3.5 kDa membrane (Cellu Sep Membrane, Seguin, TX, USA) in order to remove low molecular weight components. The resulting product was polymerized with glutaraldehyde and extensively dialyzed in a 100 kDa cut‐off dialysis membrane (Millipore, Bedford, MA, USA) against bi‐distilled water to remove non‐polymerized compounds. The resultant solution was freeze‐dried, obtaining the *Alternaria* depigmented‐polymerized extract (ADP). Three different batches were manufactured in order to investigate the consistency of the methodology. All of the process was conducted under strict compliance with GMP principles and following internal procedures (Laboratorios LETI, Spain 19).

### Serum samples and PBMC

Serum samples from eight *Alternaria* sensitized individuals were purchased at Plasmalab International (Everett, WA, USA), which operates in full compliance with U.S. Food and Drug Administration regulations. All sera presented a 4 class specific IgE level against Alternaria by Immuno CAP system (Thermo Fisher Scientific, Waltham, MA, USA), being the mean value of 63.8 ± 14.5 KU/L. These sera were pooled. The resulting pool presented a positive sIgE level of 55.8 KU/L to complete *A. alternata* extract, 67.0 KU/L to Alt a 1 and negative to bromelain (0.25 KU/L). This pool of sera was used for the determination of IgE binding to the allergen (immunoblot, ELISA competition and ELISA inhibition).

Peripheral‐blood mononuclear cell (PBMC) culture supernatants from six additional *Alternaria*‐atopic donors (all sera presenting sIgE > 3.5 kU/L, mean value 33.0 ± 33.7 kU/L) not previously treated with immunotherapy, and two non atopic donors (sIgE < 0.35 kU/L) were included to evaluate the capacity of the extract to stimulate cytokine production. PBMCs were obtained from the Andalusian Health Service, Spain (approved by the Research Ethics Committee).

Polyclonal antibodies were used for IgG‐binding assays to allergen extracts. Specific antibodies were induced after the administration of NE or ADP in two New Zealand white rabbits after three immunizations with 200 μg of NE or ADP with Complete Freund's Adjuvant (first immunization) or Incomplete Freund's Adjuvant (second and third immunizations) every three weeks. All procedures were approved by Biolab Institutional Review Board (Biolab, S.L., Colmenar Viejo, Spain), and followed local ethics rules for animal experimentation.

### Allergen extract characterization

#### Protein content

The protein content of NE and ADP extracts was measured by the Lowry–Biuret method (Sigma Diagnostics, St. Louis, MO, USA) following the manufacturer's instructions.

#### Protein profile

Fifty micrograms of lyophilized NE and ADP extracts were loaded in Sodium Dodecyl Sulfate Polyacrylamide Gel Electrophoresis (SDS–PAGE) with 2.67% C, 15% T acrylamide under reducing conditions and stained with 0.5% solution of Coomassie Blue R‐250 (Bio‐Rad Laboratories, Hercules, CA, USA).

Addtionally, ADP molecular weight distribution was determined by high‐performance Size Exclusion Chromatography (SEC) using a Bio SEC‐3 Column (Agilent Technologies, Santa Clara, CA, USA) in a 1200 series HPLC system (Agilent), at 1 ml/min in 150 mM phosphate buffer, pH 7. Detection was performed at UV–215 nm and Gel Filtration Standard (BioRad) was used as molecular weight marker.

#### Allergenic profile

Electrophoretically separated bands were transferred to an Immobilon®‐P membrane (Millipore). Thereafter, the membrane was incubated overnight with the pool of sera from Alternaria‐sensitized individuals. Afterwards, the membrane was washed and finally incubated with monoclonal α‐human‐IgE‐PO (Ingenasa, Madrid, Spain). Finally the reaction was developed with Clarity™ Western ECL Substrate (BioRad).

Alt a 1 was identified by using α‐Alt a 1 monoclonal antibody labeled with biotin (BI‐3B6, Indoor Biotech, Charlottesville, VA, USA) as the primary antibody. After washing, the membrane was incubated with streptavidin‐peroxidase and finally developed.

#### Major allergen quantification

Major allergen, Alt a 1, was quantified in NE using a specific commercial kit (EL‐AA1) following the manufactureŕs instructions (Indoor). Standard data were adjusted to a four‐parameter logistic curve by the least‐squares method. Determination of Alt a 1 in ADP was estimated based on NE Alt a 1 determination and yield.

#### Biological potency

Biological potency of the extracts was calculated by ELISA competition assays, as previously described 20. Briefly, each extract was compared with the In House Reference Preparation (IHRP), previously in vivo standardized (data not shown, Laboratorios LETI). Nunc microplates (Thermo Scientific) were coated with anti‐IgE (Ingenasa). The pool of sera from *Alternaria* sensitized patients was incubated in this plate. Dilutions of the sample and IHRP were incubated with the allergen labeled with peroxidase. The mixture was added to the coated plate and incubated. Afterwards, development solution (chromogen) was added, stopped with sulfuric acid and measured at OD450 nm. The percentage of loss of potency was calculated as the difference of biological potency between NE and ADP divided by NE potency.

Results were expressed in HEP/mg, considering 10 HEP the quantity of extract that elicits a similar skin reaction as a histamine prick of 10 mg/ml.

#### Protein profile: Allergen identification

The presence of allergens in ADP was determined by mass spectrometry. Briefly, ADP extract was digested with trypsin and the peptide mixture was analyzed in a nanoACQUITY liquid chromatography system (Waters Corporation, Milford, MA, USA) coupled to a LTQ‐Orbitrap Velos (Thermo) mass spectrometer. Raw data were collected with Thermo Xcalibur software v.2.2 (Thermo). A database search was performed through the Mascot search engine using Thermo Proteome Discover against the Uniprot database 21.

### In vitro toxicity

#### Bacterial reverse mutation test (Ames test)

The test was performed in accordance with OECD Guideline 471 for the Testing of Chemicals (Bacterial Reverse Mutation Test. Adopted 21st July 1997) and the test Method B13/B14 of Commission Directive 2000/32/EC.

Briefly, five bacterial strains were exposed to five concentrations of ADP (5.00–0.06 mg/plate) with and without metabolic activation system (post mitochondrial supernatant S, Molecular Toxicology Inc., Boone, NC, USA) under the direct incorporation and the pre‐incubation procedures. Plates were incubated for 48 h at 37°C and colonies were counted. In the direct incorporation procedure the mixture was immediately poured over a minimal agar medium plate and incubated at 37°C for 48 h. Whereas in the pre‐incubation procedure, the mixture was incubated for 20 min at 37°C prior to being poured over the minimal agar medium plate. Vehicle was used as a negative control.

The number of revertant colonies per plate was counted and recorded by an automatic colony counter.

### In vivo toxicity

#### Abnormal toxicity

The abnormal toxicity test of the extracts was performed according to European Pharmacopoeia 8th Ed. 22. A total of 1 ml of 2 mg/ml ADP was intraperitoneally injected to two guinea pigs and five mice and they were observed during 7 days. All procedures were approved by the Biolab Institutional Review Board, and followed local ethics rules for animal experimentation.

#### Fish embryo acute toxicity (FET) test and limit test

Zebrafish (*Danio rerio)* was used to evaluate acute toxicity based on the OECD TG236 guideline for chemicals testing 23. This test was performed as a tentative assay to evaluate toxicity, for example due to the presence of mycotoxins. It has not previously been performed with allergens, so results could not be compared with previous data.

Firstly, a limit test of ADP was performed using 100 mg/L in water with 0.1% DMSO. A totao of 2 ml were used for incubating each fertilized egg at 26°C during 24, 48, 72, and 96 h. Negative controls (water and vehicle) and positive mortality control (3.7 mg/L of 3, 4‐dicloroaniline) were also used. Twenty eggs were used for the treatment group, positive and vehicle controls, and four eggs for water control as internal plate control on each of the above plates. Mortality, sublethal effects, teratogenic effects, and hatching percentage were recorded after 24, 48, 72, and 96 h.

Secondly, taking into account limit test results, the FET test was performed using 2000, 909, 413, 188, and 85 mg/L doses of ADP. Water, vehicle and positive controls were also included. Ten eggs were used in each treatment group, positive and vehicle controls, and two eggs for water control as internal plate control. Mortality, sublethal effects, teratogenic effects, and hatching percentage were recorded after 24, 48, 72, and 96 h.

Animal care was carried out by qualified technicians supervised by veterinarians. Animals were treated in accordance with Spanish and European laws (Royal Decree 53/2013 and Directive 2010/63/EU) and the International guidelines for ethical conduct in the care and use of experimental animals were applied throughout the study.

#### Maximum tolerated dose (MTD) and dose‐range finding study

ADP extract was adsorbed onto aluminum hydroxide 0.3% to evaluate the safety of the final formulation in vivo. A first phase was performed in order to identify MTD after a single subcutaneous administration to Sprague–Dawley rats. Two male and two female rats were included in each treatment group: 0 (vehicle), 0.047, 0.47, and 4.7 mg per kg (of animal) in a dose volume of 0.5 ml. The MTD was identified as the dose that produced neither mortality nor more than a 10% decrement in body weight nor clinical signs of toxicity.

In the second phase, animals were daily administered for 7 days with the MTD or the vehicle. Five male and five female rats were included in both groups. Safety assessment relied on body weight, food consumption, local, and general clinical observations recorded over the 7‐day dosing period, together with clinical pathology and gross necropsy evaluation performed upon completion of the dosing period. Unpaired *t*‐test was performed as statistical analysis.

All procedures were approved by the Vivotecnia Institutional Review Board (Vivotecnia, Tres Cantos, Madrid, Spain), and followed local ethics rules for animal experimentation.

### In vitro efficacy

#### IgE blocking by IgG antibodies

The capacity of ADP and NE to induce allergen‐specific polyclonal IgG antibodies able to block IgE binding sites to the allergen was evaluated by ELISA inhibition, as previously described 24. Briefly, microplates were coated with NE (2 μg/well). After incubation with rabbit polyclonal antibodies generated against ADP or NE, plates were incubated with the pool of sera from Alternaria‐sensitized individuals. A secondary antibody anti‐human‐IgE‐PO (Southern Biotech, Birmingham, USA) was used for determination of Optical Density (O.D.) at 405 nm. Percentage of IgE‐inhibition was calculated by comparing IgE binding after incubation with preimmune sera or final bleeding polyclonal antibodies against NE or ADP, according to the formula: % inhibition = [100‐(preimmune O.D./final bleed O.D.)*100].

#### Cytokine production

Cytokine content in supernatants was measured by ELISA‐based Milliplex® Mag Human kit (Millipore) performed in accordance with the manufacturer's instructions. Briefly, PBMCs (8.5 × 10^5^ cells per well) from donors (six atopic and two non atopic controls) were stimulated in duplicate with NE or ADP extract (50 µg/ml), and the production of IFN‐γ, IL‐4,IL‐6, IL‐10, and TNF‐α cytokines was measured by duplicate in culture supernatants after 72 and 120 h of incubation at 37°C in 5% CO_2_ atmosphere. Culture medium RPMI‐1640 (Sigma–Aldrich, St. Louis, MO) and concanavalin A (Con A, 5 μg/ml) were used as negative and positive controls, respectively. Cell viability was determined using trypan blue as vital stain and Cell Countess counter (Invitrogen, Carlsbad, CA). Supernatant was collected and quantified using Milliplex® Mag kit. Standard data were adjusted to a five‐parameter logistic curve. *T*‐student statistical analysis was performed (normal distribution data). A *p*‐value <0.05 was considered statistically significant.

## Results

### Allergen extract characterization

Three batches of ADP were manufactured from their corresponding NE extracts. Mean protein content of the three NE batches was 46 ± 12.5 μg protein/mg lyophilized extract, and 118 ± 25.7 μg protein/mg lyophilized extract for ADP. Alt a 1 content was 4.1 ± 0.43 μg/mg lyophilized NE and 25.3 ± 2.1 μg/mg lyophilized ADP. Mean biological potency of NE was 29 ± 13.5 HEP/mg, and residual potency in polymer was 0.04 HEP/mg. Therefore, there was a 99.8% reduction of the IgE binding capacity in ADP with respect to NE.

**Figure 1:**
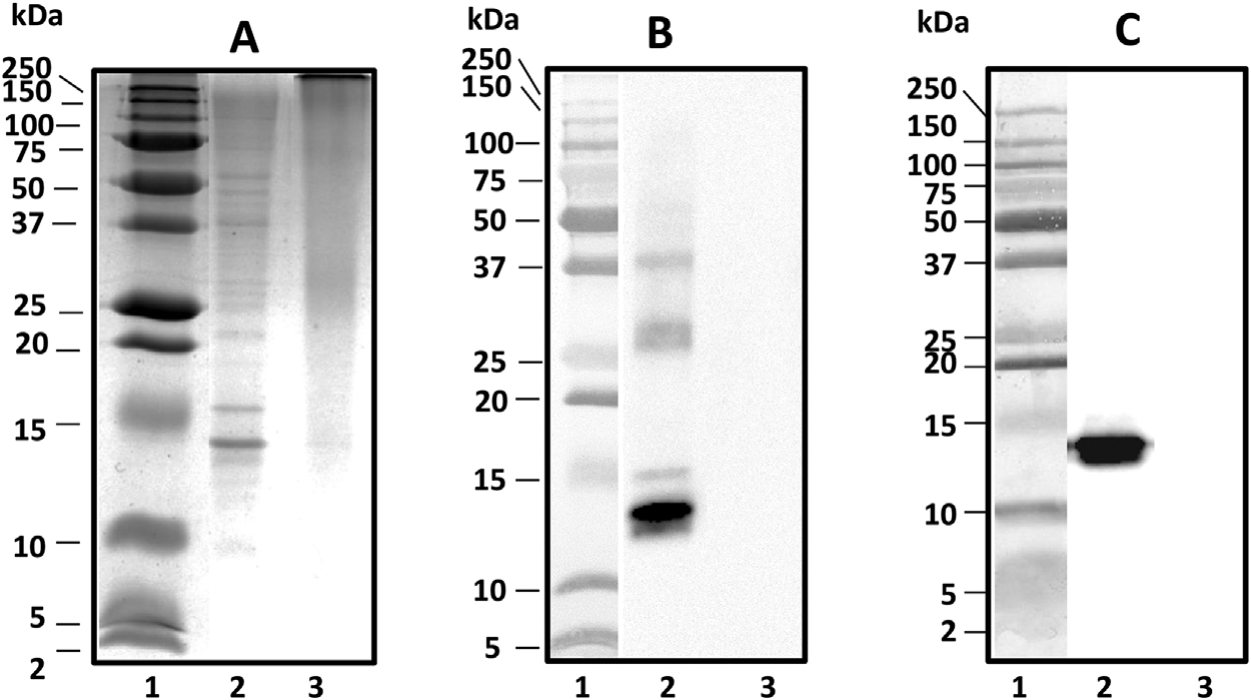
SDS–PAGE (A) and immunoblot (B and C) of *Alternaria* under reducing conditions (15%T–2.67%C): Precision Plus Protein Dual Extra Standard (lane 1), NE (50 μg extract, lane 2), and ADP (50 μg, lane 3). Immunoblots were performed using serum from *Alternaria* sensitized patients (B) or monoclonal antibody α‐Alt a 1 (C). ADP: *Alternaria* depigmented‐polymerized extract; NE: *Alternaria* native extract.

The most intense protein band was observed at 13 KDa in NE (Fig. 1), being also the most intense band in immunoblots using patient́s IgE. Its identity as Alt a 1 was confirmed using monoclonal antibodies. Other protein bands at 15, 27, and 38 KDa were also observed.

**Figure 2:**
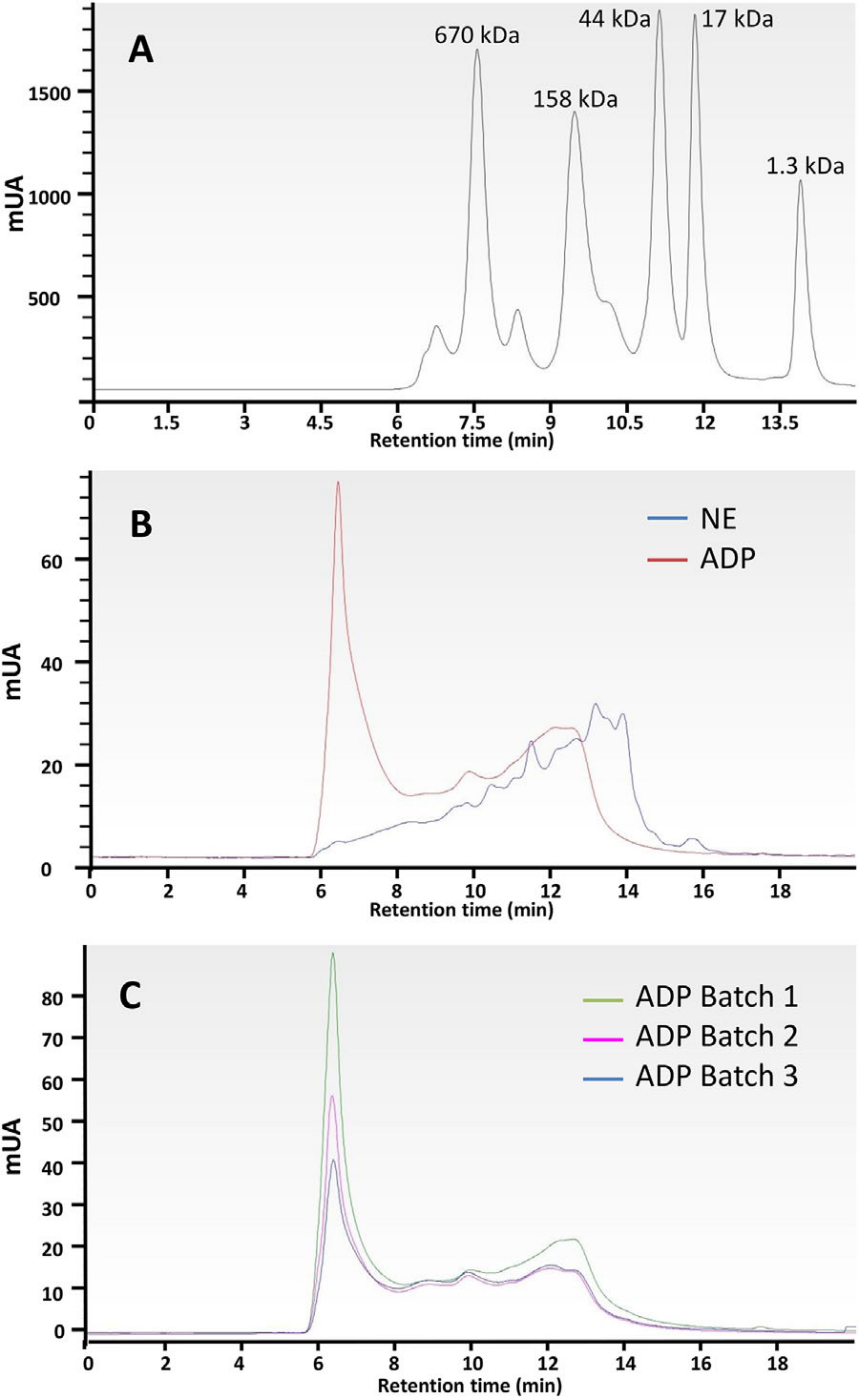
Size exclusion chromatograms. (A) Gel Filtration Standard. (B) comparison of NE (blue line) and its ADP (red line) detected at 215 nm. C, comparison of three different ADP profiles. Elution time in minutes. ADP: *Alternaria* depigmented‐polymerized extract; NE: *Alternaria* native extract.

Molecular weight distribution, determined by HPLC, showed a significant modification of the chromatographic profile of ADP with respect to NE consistent with an efficient protein polymerization (Fig. 2). NE showed main peaks of 4–40 KDa, while ADP presented a predominant peak higher than 1500 KDa. This chromatographic profile showed high consistency among the different ADP batches.

Finally, the presence of allergens was confirmed in the ADP. Mass spectrometry was performed in ADP, identifying sequences of Alt a 1, Alt a 3, Alt a 6, and Alt a 8. Sequence coverage compared to Uniprot sequences were 40% for chain 1 Alt a 1 (code P79085), 27% for Alt a 3 (P0C0Y4), 10% for Alt a 6 (D2J4C1), and 12% for Alt a 8 (Q9HDT3).

### In vitro toxicity

The Ames test showed that ADP did not induce point mutations or frame‐shifts in the genome of the bacterial strains with or without metabolic activation regardless of the procedure. Therefore, it was considered non‐mutagenic nor pro‐mutagenic under the experimental conditions assayed (exposure dose range of 5.00–0.06 mg/plate).

### In vivo toxicity

In the abnormal toxicity test, all the animals survived and none of them showed any signs of ill health or weighed less at the end of the test period than at the start, so ADP passed the test.

Regarding the zebrafish embryos tests, an initial limit test showed that a concentration of 100 mg/L did not present any mortality, sublethal effects nor teratogenicity after 96 h, while hatching percentage was 100%, showing the biosecurity of the product. Negative and positive controls met the Test Guideline 236 criteria 23.

**Figure 3:**
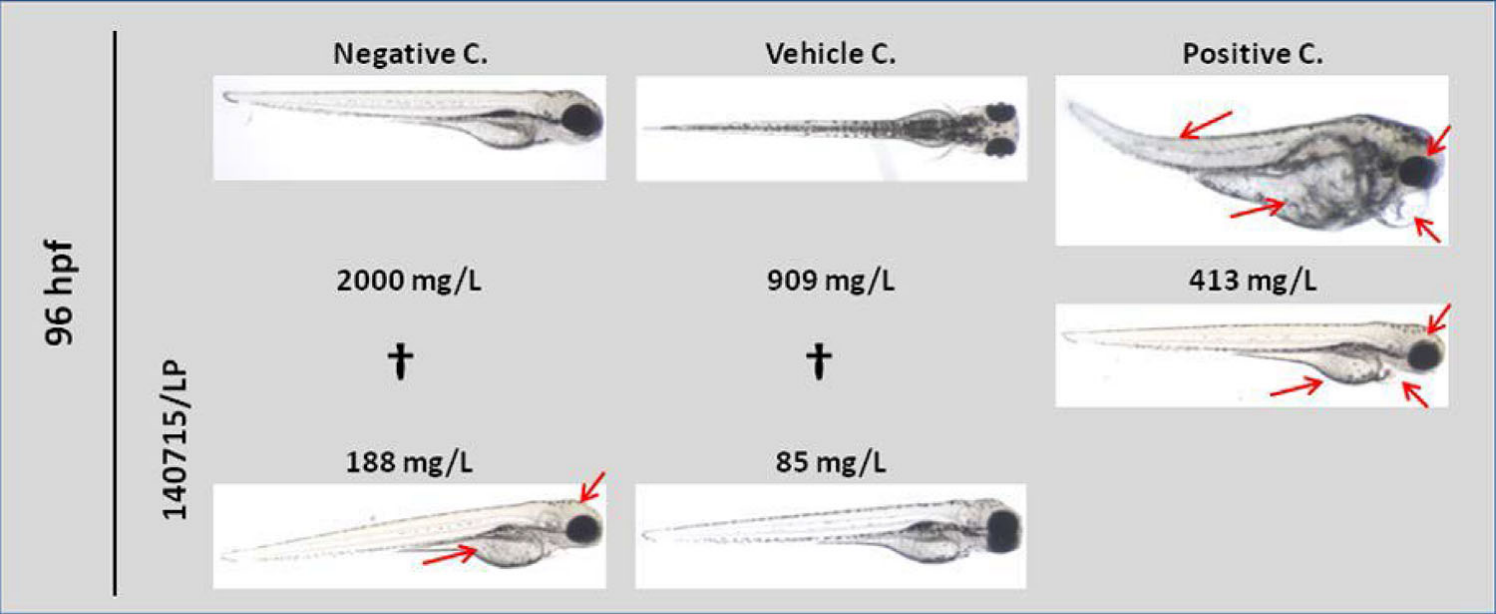
A total of 96 h post fertilization (hpf) representative images of zebrafish embryos exposed to different concentrations of ADP. Control groups are also shown. Red arrows highlight embryo defects. (†: 100% mortality). ADP: *Alternaria* depigmented‐polymerized extract.

In spite of the absence of mortality in the limit test, an acute toxicity test was performed using serial dilutions from 2000 to 85 mg/L of ADP (Fig. 3). Mortality reached 100% at 48 h in 2000 and 909 mg/L doses, while it was 30% at 96 h in the 413 mg/L treatment. However, sublethal effects were observed in 100% of the eggs at 48h in 413 mg/L, and teratogenic effects in 100% at 48 h in 188 mg/L. Hatching rate was over 80% at 188 and 85 mg/ml. Consequently, mean Lethal Concentration (LC_50_) was determined at a concentration between 413 and 909 mg/L, the Lowest Observed Effect Concentration (LOEC) was 188 mg/L, and the No Observed Effect Concentration (NOEC) was 100 mg/L.

Maximum Tolerated Dose (MTD) and dose‐range finding study were performed in rats. MTD was initially evaluated. Doses of 0, 0.0125, 0.125, or 1.25 mg were administered subcutaneously, and animals were observed during 7 days. Neither erythema nor edema was observed at the injection site in any animal from any experimental group, and the only local abnormality recorded was the presence of induration at the injection site in mid and high dose groups (0.125 and 1.25 mg/animal, respectively). Upon necropsy, performed on day 8 of the observation period, the presence of subcutaneous nodules at the inoculation site was found in all animals (control and treatment groups). According to these results, the high dose level tested (1.25 mg/animal) was selected as the MTD to be evaluated in the confirmatory phase.

In the confirmatory phase, rats were daily administered with MTD or the vehicle during 7 days. No treatment related mortality, systemic clinical signs nor effect on food consumption, were recorded in response to treatment with ADP at MTD. The only local reaction was the presence of induration at the injection site, which was found in most animals. Test item treated females showed a statistically significant increase in absolute body weight gain by the end of the treatment. Relative neutrophil count (%) and absolute neutrophil count were found to have increased in females (statistically significant) and males (without reaching statistical significance when compared to the control group) from the ADP treated group (1.25 mg/animal/day). Besides, ADP treatment led to a decrease in albumin concentration (statistically significant) accompanied by an increase in globulins levels (statistical significance only in males), always compared to control group.

### In vitro efficacy

Two different approaches were used to evaluate in vitro efficacy. The first method was to evaluate the capacity to induce blocking IgG antibodies. The second method was to evaluate the capacity of the extracts to induce cytokines production.

**Figure 4:**
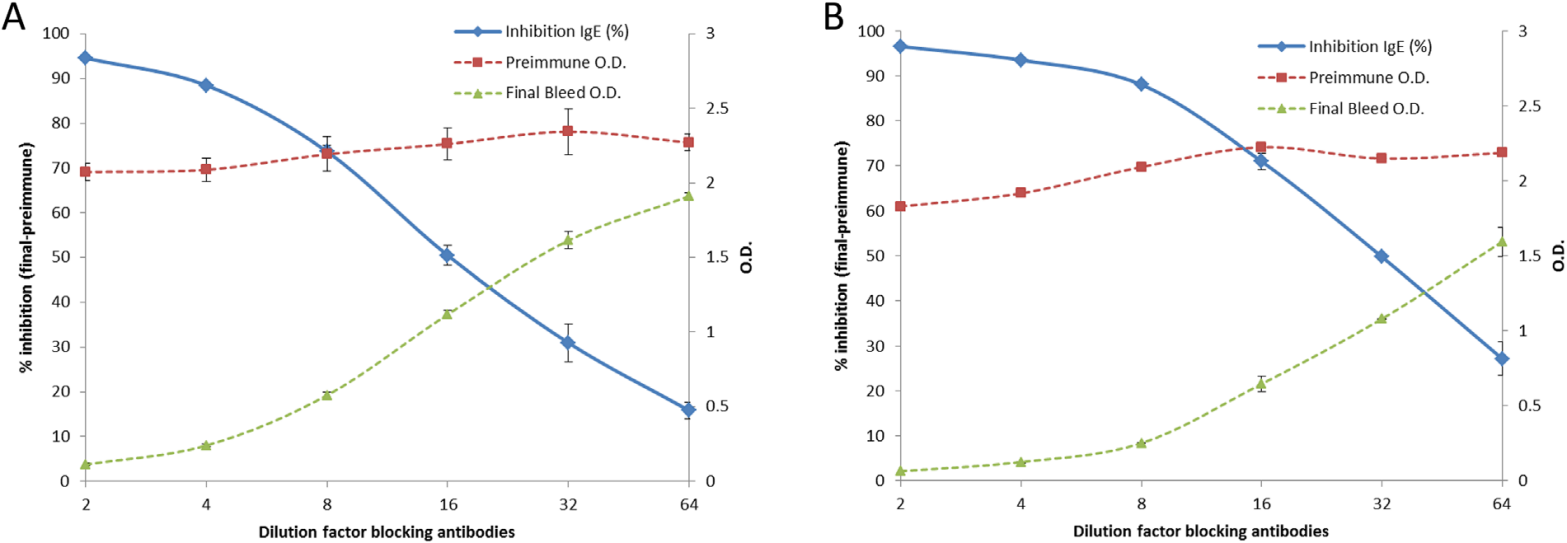
Blocking IgG antibodies. The graph shows the titration of human specific IgE binding (right y axis: O.D.) after inhibition with preimmune or final bleed sera from rabbits immunized with NE (A) or ADP (B). The percentage of inhibition obtained at different dilution factors is represented by the continuous line (left y axis). Experiments were performed by duplicate.

ADP and NE extracts induced the production of IgG antibodies able to block IgE‐binding sites of NE in a similar way. However, percentages of inhibition obtained for the ADP extract were higher at all dilution factors than the ones obtained for the antibodies induced by NE (Fig. 4).

**Figure 5:**
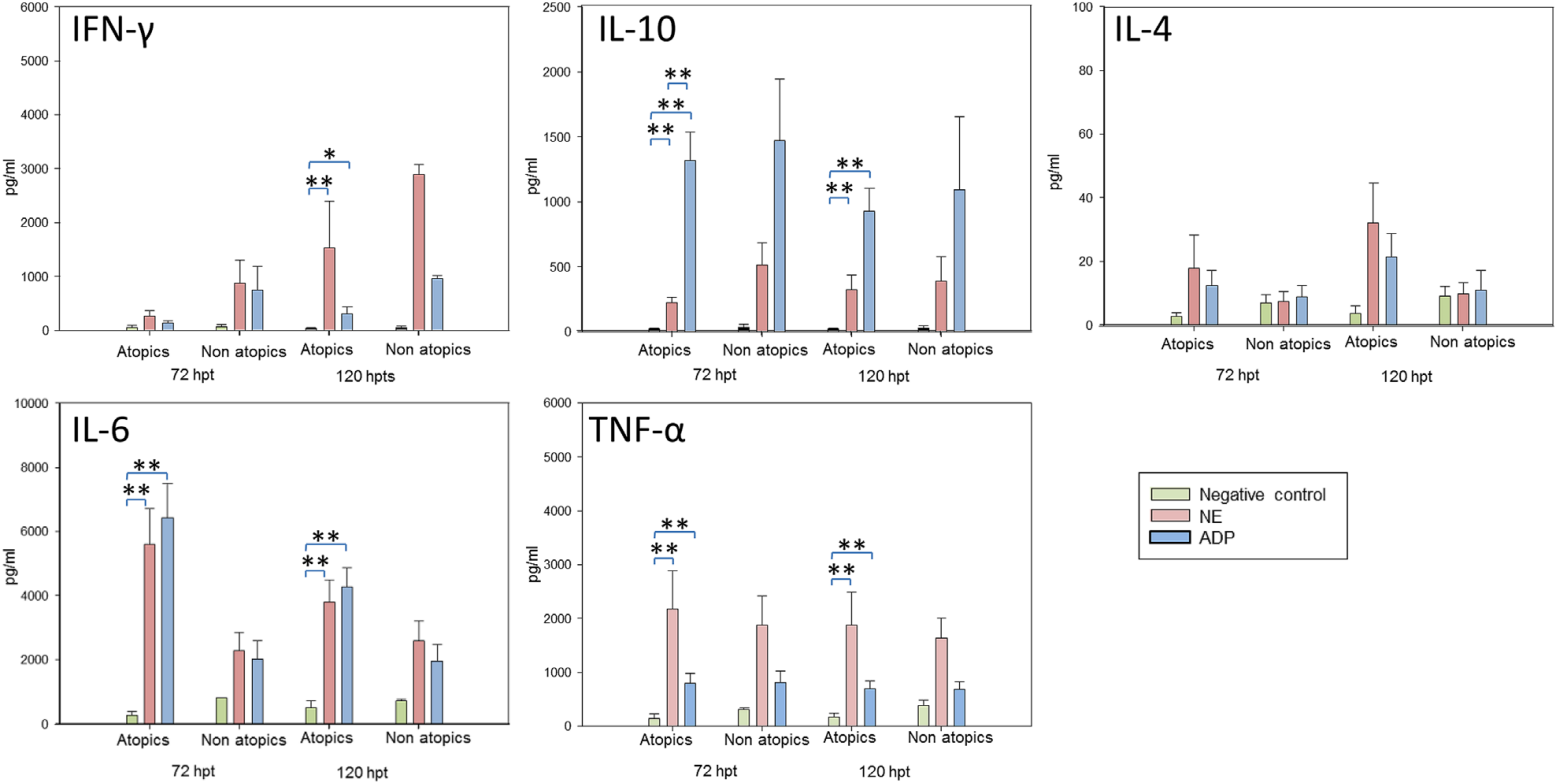
Mean cytokine production by PBMCs from atopics (*n* = 6) or non atopics (*n* = 2) donors after stimulation by duplicate with negative control (green), NE (pink), ADP (blue) at 72 or 120 h post treatment (hpt): IFN‐γ, IL‐10, IL‐4, IL‐6, TNF‐α. Error bars refer to standard deviation. * Refers to *p *< 0.05, **p < 0.01 (*t*‐student). ADP: *Alternaria* depigmented‐polymerized extract; NE: *Alternaria* native extract.

The capacity of NE and ADP to stimulate PBMC to produce IFN‐γ, IL‐4, IL‐6, IL‐10, and TNF‐α was evaluated in atopic and non‐atopic donors (Fig. 5). Cell viability was over 80% in all groups 120h post treatment (hpt), which shows that there was no toxic effect in any of the treatments.

IFN‐γ and IL‐4 production was higher at 120 hpt than at 72 hpt, while IL‐10 and IL‐6 levels decreased at longer periods. IFN‐γ, IL‐10, IL‐6, and TNF‐α production was significantly higher at 120hpt with both NE or ADP than negative control in PBMCs from atopic patients (*p* < 0.01, excepting for IFN‐γ induction by ADP versus negative control: *p* < 0.05). IL‐10 was significantly higher in ADP treatment of atopics versus NE treatment at 72 hpt (*p* < 0.01).

Atopics and non‐atopics were also compared. Differences in cytokines production were observed in IL‐10 induction with NE at 72 hpt (higher in non‐atopics, 226 vs 514 pg/ml) and IFN‐γ induction with ADP at 120 hpt (higher in non‐atopics, 309 vs 955 pg/ml).

Concavanalin A was used as a positive control (data not shown). Results were in all cases significantly higher than in negative control (vehicle) (*p *< 0.05) except for IL‐4 production 120 h after treatment in non‐atopic PBMCs (*p *> 0.05).

## Discussion

It has been clearly demonstrated that fungi constitute an important allergenic source 25. Apart from the elimination and avoidance of the offending allergen, which is not possible for outdoor allergens, the only treatment able to modify the course of the allergic disease is AIT. In the case of *Alternaria* allergy, due to its relation with asthma severity 15, specific immunotherapy with allergenic extracts can be of significant benefit 16. Native extracts consisting of a mixture of allergens have been traditionally used in *Alternaria* immunotherapy 17, 18, though, recently, a purified Alt a 1 vaccine has demonstrated its capacity to induce sIgG against Alt a 1 26. With the objective of reducing adverse events, other approaches such as the use of an Alt a 13 hypoallergen and Alt a 1‐derived peptides are under evaluation 27, 28. As an alternative, the use of chemically modified allergoids has been released but until now no reports demonstrating the efficacy or safety have been published. However, the evidence of efficacy and safety of this kind of treatment with other respiratory allergens has been demonstrated so far 29.

With this objective, we developed a new *Alternaria* allergoid for AIT based on the concept of depigmentation and glutaraldehyde polymerization. The results obtained indicated that the manufactured extracts showed a reduction in IgE binding capacity (>99%) indicating a high degree of polymerization, respect to their corresponding NE. The consistency of the method was demonstrated by manufacturing three different batches from three different lots of raw materials. The presence of relevant allergens in ADP, including Alt a 1, which is crucial for the efficacy of AIT, was also confirmed. Furthermore, the product presented a lower IgE binding capacity than conventional native products.

Regarding molds, toxicity is a critical issue as a consequence of the possibility of production of mycotoxins and ochratoxins. FET test is a method recommended to determine acute toxicity of chemicals on embryonic stages of fish 23, and in that sense, the toxicity of mycotoxins has been previously investigated in zebrafish models 30. The results of the FET assay with the *Alternaria* extract showed a LC_50_ higher than 100 mg/L, which is the threshold concentration of toxicity for chemicals. However, as far as we know, this is the first time that this assay has been performed to evaluate the toxicity of allergen extracts, and no reference values have been established for these kinds of products.

Additionally, single dose toxicity in rats was evaluated and a maximum tolerated dose was established at 1.25 mg ADP/day. Subcutaneous administration of ADP at MTD during 7 consecutive days was well tolerated. Injection site reactions were observed and attributed to the aluminum hydroxide as reported with other vaccines containing this adjuvant 31. Some hematological alterations were detected such as an increase in blood neutrophil count and globulin, and a decrease in albumin. These alterations can be considered indicators of inflammation induced by the vaccine. Similar results have been found in other repeated dose toxicity studies of allergen products though with different adjuvants 32. Abnormal toxicity test was performed with a concentration that exceeded MTD, and no signs of toxicity were observed. This test is utilized as a quality control release test when allergen extracts are obtained from molds and intended for parenteral administration 33. However, its usefulness as a predictor of toxicity is questioned and recommendations to be removed have been made 34.

Finally, though genotoxicity is not usually tested in vaccines, the Ames test was performed as a preliminary evaluation of the effect of the extract chemical modification, showing that no mutagenic effect was observed for ADP.

Regarding efficacy issues, AIT lacks confident biomarkers or immuno‐chemical parameters which may predict the success of the products. The concept of AIT, its target and advances in the knowledge of the mechanisms of action, have established that AIT should promote a cellular and a humoral immune response. In that sense, AIT should have the capacity to induce changes in the polarization of allergen‐specific T cells (cellular immune response) and an increase in the production of blocking antibodies against offending allergens, among other effects (humoral immune response 35). Additionally, AIT induces regulatory T cells (Treg) that inhibit T‐helper 2 (Th2) cell proliferation in an IL‐10 and TGF‐β‐dependent response, and an increase in IFN‐γ −producing Th1 cells 36. With the objective of investigating the immunological efficacy of ADP, IFN‐γ, IL‐10, IL‐4, IL‐6, and TNF‐α induction were evaluated in PBMCs from allergic donors. One limitation of our study is the low number of samples included, though we consider that the results are representative for this preliminary proof of concept. In this case, new complementary studies should be performed in patients receiving AIT with the objective of investigating the evolution of the immunological parameters before and after treatment. In our study, the results showed an increase in IL‐10, IFN‐γ, IL‐6, and TNF‐α levels in atopic and non‐atopic patients.

IFN‐γ increases MHC‐I expression by CD8+ (cytotoxic) T cells and stimulates Th1 differentiation and increases TNF‐α synthesis, which is an inflammatory cytokine. TNF‐α and IFN‐γ presented a higher induction with NE than with ADP, which suggests a T cell shift to effector cells. Previous studies have also shown higher IFN‐γ titers in PBMCs from grass pollen and house dust mite atopic donors after stimulation with NE than after stimulation with depigmented‐polymerized extracts 37, manufactured in a similar way to ADP.

On the other hand, IL‐10 levels were significantly higher after treatment with ADP than with NE in atopic patients. IL‐10 is an anti‐inflammatory cytokine that inhibits T‐cell cytokine production and the activation of mast cells and eosinophils. However, the importance comes from its association to the induction of tolerance to allergens, linked with the role of the Tregs. Nowadays, it is recognized as the key role of Treg induction in efficacious AIT 38, and IL‐10 is the main player in the regulation, as has been demonstrated in different studies of patients treated with AIT 39. It has been suggested that IL‐10 may lead to IgG4 production by memory B cells, preferably at high allergen doses 39, 40. IgE‐mediated allergic reactions are inhibited by competitive binding of IgG4 against the allergen, which avoids IgE‐allergen presentation by B cells to specific T‐cells 41, 42, associated with long‐term clinical tolerance. This effect is related to the blocking activity of IgG rather than absolute levels of IgG4 43. The higher capacity of depigmented‐polymerized extracts, compared to native extracts, to stimulate in rabbits the synthesis of specific IgG antibodies that recognize human IgE epitopes has been demonstrated in this work, as it was observed in previous studies with other allergenic sources 24. Subtle differences observed in cytokine induction in response to stimulation with NE or ADP suggests a different route of tolerance induction. NE may favor CD4+ T cell shift to Th1 (higher IFN‐γ and TNF‐α), while ADP to Treg (IL‐10). Previous works also suggest that depigmented‐polymerized extracts induce a lower proliferation of effector T cells, which may contribute to side effects, than NE 37.

These in vitro results should be confirmed in additional assays, e.g., animal model, to acquire a better knowledge of AIT with *Alternaria* allergoids, as well as associated immunological changes and connection between cellular and humoral responses, including the determination and development of the different cellular types (especially Treg cells) involved.

In summary, we have shown the characterization of a new *Alternaria* depigmented‐allergoid, which may be suitable for AIT based on quality, consistency of the manufacturing process, and safety and efficacy of non‐clinical results. No toxicological effect was observed by different methods, and it may be safer than NE due to its lower IgE binding capacity. Furthermore, the preliminary immunological data suggest that ADP is an effective extract to be used in AIT treatment because it leads to cytokine stimulation related with the induction of immune tolerance and promotes the synthesis of IgG antibodies able to block IgE binding sites in the allergens.

## Acknowledgments

We thank Tamara Aranda and Beatriz Rojas for their participation in the technical work of the study.

## Conflict of Interest

This study was funded for Laboratorios LETI. Dr. María Morales, Dr. María T. Gallego, Dr. Víctor Iraola, Dr. Raquel Moya and Dr. Jerónimo Carnés are employees of Laboratorios LETI.
